# Detection of Bacteria-Induced Early-Stage Dental Caries Using Three-Dimensional Mid-Infrared Thermophotonic Imaging

**DOI:** 10.3390/bioengineering10010112

**Published:** 2023-01-12

**Authors:** Robert Welch, Koneswaran Sivagurunathan, Pantea Tavakolian, Kimberly Ngai, Bo Huang, Stephen Abrams, Yoav Finer, Andreas Mandelis

**Affiliations:** 1 Center for Advanced Diffusion-Wave and Photoacoustic Technologies (CADIPT), Department of Mechanical and Industrial Engineering, University of Toronto, Toronto, ON M5S 3G8, Canada; 2 Institute for Advanced Non-Destructive and Non-Invasive Diagnostic Technologies (IANDIT), University of Toronto, Toronto, ON M5S 3G8, Canada; 3 Faculty of Dentistry, University of Toronto, Toronto, ON M5G 1G6, Canada; 4 Quantum Dental Technologies, Cliffcrest Dental Office, Toronto, ON M5G 1G6, Canada; 5 Institute of Biomedical Engineering, University of Toronto, Toronto, ON M5G 1G6, Canada

**Keywords:** dental caries, (enhanced) truncated correlation photothermal coherence tomography, linear iso phase (LIOP), three-dimensional imaging, thermophotonic

## Abstract

Tooth decay, or dental caries, is a widespread and costly disease that is reversible when detected early in its formation. Current dental caries diagnostic methods including X-ray imaging and intraoral examination lack the sensitivity and specificity required to routinely detect caries early in its formation. Thermophotonic imaging presents itself as a highly sensitive and non-ionizing solution, making it suitable for the frequent monitoring of caries progression. Here, we utilized a treatment protocol to produce bacteria-induced caries lesions. The lesions were imaged using two related three-dimensional photothermal imaging modalities: truncated correlation photothermal coherence tomography (TC-PCT) and its enhanced modification eTC-PCT. In addition, micro-computed tomography (μ-CT) and visual inspection by a clinical dentist were used to validate and quantify the severities of the lesions. The observational findings demonstrate the high sensitivity and depth profiling capabilities of the thermophotonic modalities, showcasing their potential use as a non-ionizing clinical tool for the early detection of dental caries.

## 1. Introduction

Dental caries, or tooth decay, is the loss of hard tissue by acids from caries-forming (cariogenic) bacteria, and is one of the most prevalent chronic diseases in humans [1,2]. The development of caries is a result of the metabolic activities of cariogenic bacteria present in biofilm (dental plaque) adhering to the tooth. The cariogenic bacteria convert sugars present in oral environment of the host primarily into lactic acid, causing a reduction in the pH of the biofilm’s liquid phase, leading to mineral phase/crystal dissolution of tooth hard tissue [2]. For early caries, which is limited to the outmost layer of tooth structure (enamel), this reduction in pH drives the dissolution of hydroxyapatite, the primary constituent of enamel (96% by weight) [3]. This reduction in mineral content (demineralization) in enamel is the cause of early caries, and gives rise to changes in optical, thermal, and other physical properties that conventional diagnostic techniques often fail to detect.

Current clinical detection of early demineralization is conducted using both intraoral examination (visual and tactile assessment) by a trained dentist and through radiography [2]. Radiographic assessment is suitable for advanced dentinal caries and beyond, but has low sensitivity (typically below 50%) when the lesion is limited to the enamel [4]. Radiographs are primarily designed for the detection of interproximal caries (caries on the contact points with adjacent teeth). If caries develops in the grooves or fissures on the biting surface of the tooth, the thick enamel shell on the buccal and lingual of the tooth will block the transmission of the X-rays, and lesions will only be detected once they are very deep into the tooth and have a large volume. In a recent meta-analysis and systematic review, the pooled sensitivity for radiography to detect any kind of proximal lesion under clinical settings was reported to be 24%, and the specificity was 97% [4]. In addition, radiography is unsuitable for frequent use due to the health risks associated with its ionizing wavelengths [5].

For intraoral examination, a trained dentist evaluates the patient’s teeth through visual assessment, inspecting for tooth color changes caused by the high light scattering nature of lesions. Dentists also conduct tactile investigations, using a blunt probe to detect tooth texture changes. Although convenient, the intraoral exam is highly technique-sensitive and inconsistent. It is primarily based on the operating dentist’s experience, often leaving early caries undetected and without the exact extent of the severity known [5,6]. Clearly, there is a need for the development/exploration of highly sensitive, reliable, and safe diagnostic tools that are capable of detecting dental caries early in its formation, which will help clinicians arrest and reverse the progression of lesions by nonsurgical intervention [7].

Near-infrared (NIR) photothermal (PT) imaging shows great promise in this field due to the co-operative optical and thermal properties contributing to signal/image generation and processing, which result in superior dynamic range, capturing the early formation of caries. In this spectral range, the optical absorption (µ_a_) and scattering (µ_s_) coefficients of healthy enamel, the outermost layer of teeth, are low, and its thermophysical properties, primarily thermal diffusivity, and conductivity are high, thereby enabling deep optical penetration for imaging of defects, accompanied by low surface heat generation. Repeated exposure to bacteria-derived acids demineralizes tooth enamel, forming micropores that cause an increase primarily in the scattering coefficient [8,9], leading to a decrease in photon penetration depth and subsequent photothermal heat generation closer to the enamel surface in caries lesions relative to the healthy surrounding enamel. In addition, the thermal diffusivity change (decrease) of caries lesions compared to that of healthy enamel [9,10] amplifies the sensitivity of photothermal modalities to the detection of early-stage caries.

Truncated correlation photothermal coherence tomography (TC-PCT) and enhanced truncated correlation photothermal coherence tomography (eTC-PCT) are two photothermal imaging modalities developed at the Center for Advanced Diffusion-Wave and Photoacoustic Technologies (CADIPT). These methods have given rise to thermophotonic imaging (TPI), an emerging PT diagnostic modality [11] currently being explored for early caries diagnosis. TPI involves the detection of photothermal waves through emitted infrared (IR) photons (Planck radiation) from hard and soft tissues using mid-IR cameras. The underlying signal generation physics and processing have already been covered extensively [11,12,13]. Therefore, only a brief overview of TC-PCT and eTC-PCT and the amplitude and phase channels will be given here. Both algorithms are pulse-compression- and matched-filter-based. They operate by correlating the in-phase (IP) reference signal, which is the binary equivalent of the chirped optical excitation signal, and a quadrature (Q) reference signal, which is a 90º phase-shifted copy of the IP signal, with the sample’s temporal (photo)thermal transients. Their main difference involves the treatment of the thermal transients prior to the matched filter. The TC-PCT algorithm truncates the thermal-transients using the IP reference signal to improve the localization of thermal transients in time and by extension depth, whereas the eTC-PCT uses the full-length thermal transients with no truncation. This also affects the sampling of the IP and Q correlation amplitudes, further enhancing the difference between the two modalities, which is discussed in greater detail elsewhere [13]. The ultimate result is that the Q correlation amplitude does not contribute to the TC-PCT amplitude channel, whereas it does for the eTC-PCT channel. The IP and Q correlation signals contribute to both the eTC-PCT and TC-PCT phase channels, but the thermal transient truncation used by TC-PCT results in low signal-to-noise (SNR) reconstructions, making it unsuitable for the biological domain where strict low-energy limitations regarding the use of lasers are adhered to.

eTC-PCT has already demonstrated excellent promise as an effective non-ionizing modality for the early detection of dental caries [14,15]; however, TC-PCT has not yet been applied to dental imaging. In addition, a new phase channel, termed the TC-PCT linear iso phase (LIOP), was recently introduced [13], and is untested in the application of caries detection. The LIOP channel was introduced to overcome the low SNR of the TC-PCT phase channel, making it suitable for use in the biological domain [13]. It differs mainly during the sampling procedure of the IP and Q correlation amplitudes, to ensure that non-zero IP and Q signals always contribute to the phase, which often does not happen in the TC-PCT phase.

Hence, the aim of this work was to evaluate the sensitivity and three-dimensional reconstruction capabilities of the TC-PCT, eTC-PCT, and the TC-PCT LIOP algorithms for the detection of bacteria-induced dental caries (formed using the protocol described in Section 2.1) in human teeth. The lesions were created using highly cariogenic bacteria species to closely mimic the formation of natural early caries. The severities of the produced caries lesions were validated using visual assessment by a practicing dentist (described in Section 2.3) and through micro-CT (µ-CT) (Section 2.4) as the non-destructive gold standard for laboratory caries detection [16]. A limited number of samples (*n* = 5) were treated and analyzed due to the exploratory nature of this study. In practice, this limits the clinical applicability of this study as statistically relevant conclusions cannot be drawn. However, the intention of the study was to showcase preliminary findings to motivate future studies to investigate the clinical relevance of non-invasive thermophotonic imaging modalities.

## 2. Materials and Methods

### 2.1. Caries Creation Protocol

To investigate the detection sensitivity of the eTC-PCT, TC-PCT, and TC-PCT LIOP algorithms to the presence of dental caries, an in vitro approach was adopted to demineralize the samples in a controlled manner. All samples were collected according to the necessary ethics requirements and considerations, which are outlined in the Institutional Review Board Statement at end of the article. The chosen bacteria-based protocol closely mimicked the natural dynamics of caries formation by lactic-acid-producing cariogenic bacteria. Initially developed by Klein et al. [1], the protocol was later adapted [17]. Five extracted human third molars were first coated using acid-resistant nail polish, except for at a ~3 × 5 mm window on three or four of their smooth surfaces to allow for direct bacteria exposure. The coated teeth were then sterilized using a 25 kGy dose of gamma irradiation (Gamma Cell, type G.C. 220). Primary cultures of Streptococcus mutans (UA159) and Lactobacillus rhamnosus (ATCC 11981) were grown separately from frozen stock in TSB-YA (5% CO_2_ at 37 °C) for 18 h. These two species were chosen due to their demonstrated cariogenic capabilities [18,19,20,21] and the increased cariogenic capabilities of multispecies biofilms over single-species biofilms [22]. The samples were then aseptically transferred into a 225 mL flask containing trypticase soy broth–yeast extract (TSB-YE) which was supplemented with 1% glucose and 2% sucrose. After this exposure, the flask was inoculated with 0.5 mL of each primary culture (5% CO_2_ at 37 °C). This procedure was repeated every 48 h of incubation with fresh primary cultures until the total exposure time (2, 4, 6, or 8 days) was reached, to maintain a high concentration of live cariogenic bacteria. This exposure was intended to generate localized bacteria-induced caries lesions varying in severity on the smooth surfaces of the samples. The teeth were subsequently removed from the flask and rinsed using a phosphate buffer solution (PBS), followed by an acetone wash to remove the nail polish from the samples. The samples were lightly brushed using a toothbrush and distilled water to ensure complete removal of residual nail polish and biofilm. They were then stored in distilled water to maintain the hydrated state of the tooth. Alternative common storage solutions such as PBS or thymol solution were not used as their effect on the photothermal response was unknown.

### 2.2. Thermophotonic Imaging Instrumentation and Parameters

**Figure 1:**
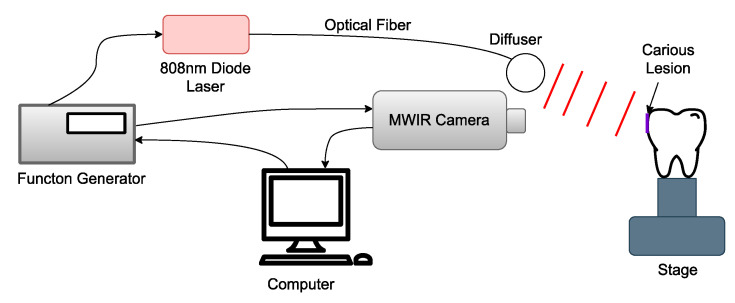
Block schematic of the thermophotonic imaging system: the bacteria-induced caries is highlighted by the purple rectangle and is positioned normal to the MWIR camera.

The imaging system shared by the TC-PCT and eTC-PCT algorithms is shown in Figure 1. It consists of a 3–5 µm spectral band camera (A6700sc, FLIR, Wilsonville, OR, USA) used to record the photothermal evolution of the sample, and a pulsed 808 nm diode laser (Jenoptic JOLD-120-QPXF2P) as a pulse chirp excitation source [23]. A computer running a LabVIEW program sends the chirped waveform to the function generator (Keysight 33500B, Colorado Springs, CO, USA) used to control the laser driver (PCO-6131, Directed Energy, Fort Collins, CO, USA), which drives the laser. The function generator also triggers the mid-wave infrared (MWIR) camera to begin recording at the correct instant. The camera uses a frame rate of 104 Hz with a frame size of 320 × 256 pixels. For analysis, the pixels are averaged by their four neighboring pixels to produce a final frame size of 80 × 64 pixels. To record the optical excitation waveform, a high-speed data acquisition module (PCI-6281, National Instruments, Austin, TX, USA) is used, which feeds the signal back to the computer. The laser uses an optical fiber connected to a collimator (F22SMA-B, Thorlabs Inc., Newton, NJ, USA), followed by a diffuser (2.5 cm, 30 degrees, Circle pattern ED1-C20, Thorlabs Inc., Newton, NJ USA) to homogenize the optical fluence. After data acquisition, a LabVIEW program implements the eTC-PCT and TC-PCT algorithms, while a separate Python program calculates the TC-PCT LIOP algorithm.

For all photothermal imaging presented in this paper, a 0.2–0.6 Hz, 12 s chirp waveform with 63 W peak power and 2.2 cm beam diameter with 40 ms pulse widths was used. This resulted in a pulse energy of 0.66 J/cm^2^ or 82% of the maximum permissible energy, making the power level safe for clinical use based on the maximum permissible energy (MPE) exposure limits on skin [24]. This chirp frequency range and duration was experimentally found to produce optimal reconstructions while minimizing imaging time. Both the low power level and short image acquisition time (12 s) were purposefully chosen to ensure that no limitations were in place preventing the potential clinical adaptation of this technology.

### 2.3. ICDAS Visual Assessment of Caries

One of the most common methods for dental carries characterization is through visual assessment by a trained dentist. The International Caries Detection and Assessment System, version 2, (ICDAS) is a common classification scale used to assess caries severity using visual inspection of the teeth in both a hydrated state and after 5 s of air drying [25]. The criteria are summarized in Table 1. All treatment regions were examined by one of the authors (S. Abrams, a practicing dentist trained in the ICDAS ranking system) prior to the thermophotonic and µ-CT imaging. Treatment areas on samples M2, M4, and M6 exposed for 2, 4, and 6 days were scored as ICDAS 0. Samples B8 and M8 (both 8 days of treatment time) developed visible ICDAS 2 caries lesions.

### 2.4. Micro-CT Imaging

In addition to ICDAS, μ-CT (SkyScan 1172, 10 W and 10 µm resolution) was employed as an additional form of caries validation. All volume reconstructions displayed were created using ImageJ [27], with the 1D luminance function manually adjusted to allow for clear visualization of lesions when present. The xy (axial) planes for reconstructions and images had a 10 µm resolution, while the z axis for volume reconstructions was displayed using a 30 µm resolution. Approximate lesion depths were measured using the mean intensity of the caries over their 2D transverse plane profile.

## 3. Results and Discussion

### 3.1. Sample B8: 8-Day Bacterial Demineralization Treatment

**Figure 2:**
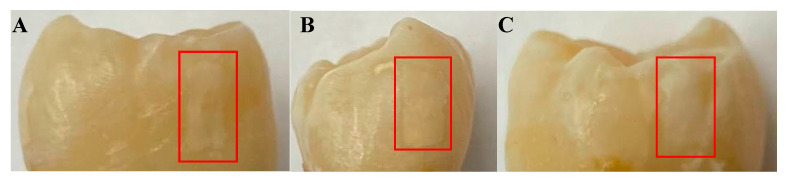
Optical images of the (**A**) buccal, (**B**) distal, and (**C**) lingual surfaces of sample B8 after 8 days of cariogenic biofilm exposure. The lesions formed by the cariogenic biofilm exposure are outlined by the red rectangles.

**Figure 3:**
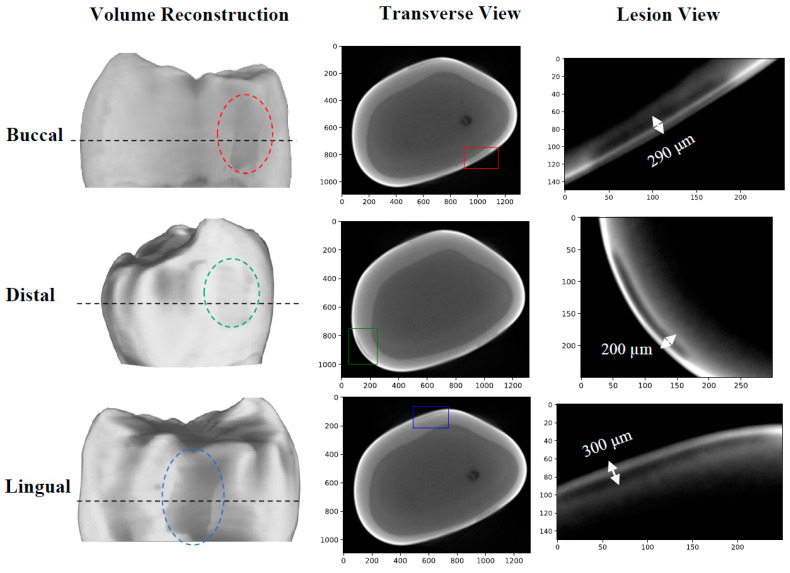
µ-CT images of the B8 sample after 8 days of cariogenic biofilm exposure: Three lesions are visible, one on each of the buccal, distal, and lingual surfaces. The dashed black line corresponds to the cross-sectional plane shown in the transverse views. The colored rectangles on the transverse views outline the position of the lesions. The “Lesion View” column shows an enlarged view of the lesions. To increase the visibility of the lesions, an adaptive histogram equalization was used [30] to improve the image contrast and visualize the edges of the lesion more definitively.

The first sample, B8, was treated for a total of 8 days in three regions: one ~3 × 6 mm region on each of the buccal, distal, and lingual surfaces. The bacterial exposure resulted in three ICDAS 2 caries lesions at the treatment regions, all of which were detected by μ-CT imaging, with the optical images shown in Figure 2 and the μ-CT results shown in Figure 3. The lesions were measured down to depths of 290 µm, 200 µm, and 300 µm using the µ-CT results for the buccal, distal, and lingual surfaces, respectively. All lesions remained within the enamel, with no progression into the dentin, meaning that their progression was still reversible using nonsurgical options such as the application of various remineralization products [28,29], making them ideal candidates for early detection.

**Figure 4:**
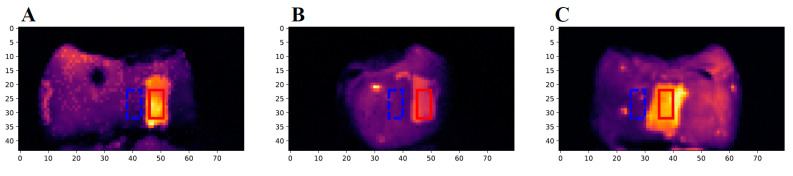
The first slices of the eTC-PCT amplitude images of sample B8: The three images show the (**A**) buccal, (**B**) distal, and (**C**) lingual surfaces of the sample. The red rectangle (10 × 5 pixels) highlights the region of the caries lesion used to determine the carious signal response of the sample, and the dashed blue rectangle (10 × 5 pixels) highlights a nearby sound region used as the reference for the sound enamel signal.

**Figure 5:**
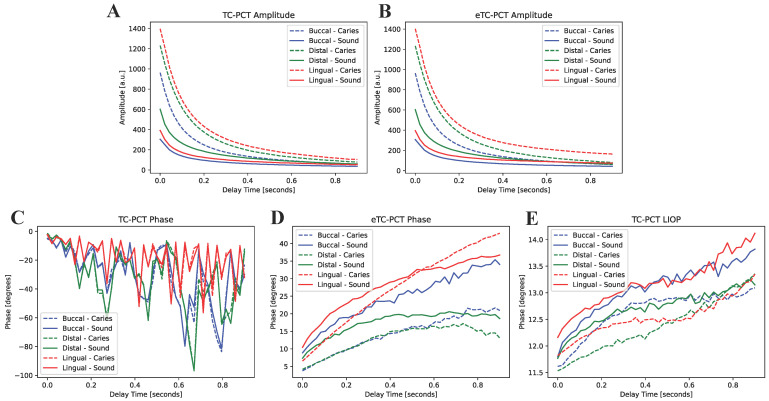
Mean amplitude and phase responses of the caries lesions and sound enamel regions highlighted in Figure 4 for (**A**) TC-PCT amplitude, (**B**) eTC-PCT amplitude, (**C**) TC-PCT phase, (**D**) eTC-PCT phase, and (**E**) TC-PCT LIOP.

The first eTC-PCT amplitude slices for each of the three caries lesions on the tooth labeled B8 are shown in Figure 4. The lesions are easily visible, and manifest themselves as large high-intensity patches. To better understand the photothermal dynamics of the lesions, the mean amplitude and phase responses of the caries lesions and sound regions (these positions are outlined in Figure 4) for each of the three surfaces are plotted and shown in Figure 5. In both TC-PCT and eTC-PCT amplitudes (Figure 5A,B), there is stronger absorption in the three lesions relative to their sound enamel references. After absorption, the thermal signals decay monotonically, indicating no obvious sign of significant subsurface absorption or changes in thermal impedance. The TC-PCT phase plots (Figure 5C) show very little difference between the caries lesions and sound regions, which is a result of the channel’s low signal-to-noise ratio (SNR). However, for both the eTC-PCT phase (Figure 5D) and TC-PCT LIOP (Figure 5E), the initial phase lags of the caries lesions are lower than their sound enamel counterparts, indicating that the thermal-wave centroids of the caries lesions are nearer to the surface.

This is expected due to the greater photon absorption coefficients in the lesions [8,9], and is consistent with the TC-PCT and eTC-PCT amplitude results. The eTC-PCT and TC-PCT LIOPs of the sound enamel and lesions then both increase with time as their centroids diffuse deeper into the enamel and dentin.

**Figure 6:**
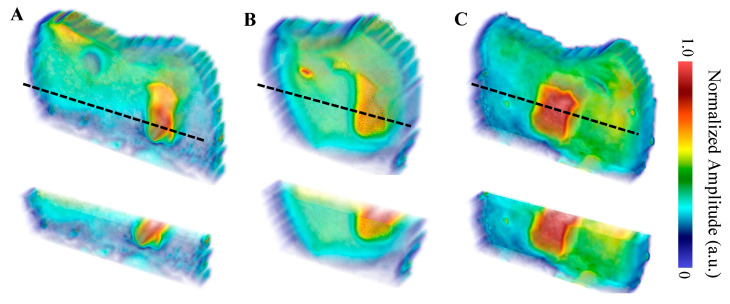
eTC-PCT amplitude reconstructions with partial transparency of the (**A**) buccal, (**B**) distal, and (**C**) lingual surfaces using a 0.2–0.6 Hz, 12 s chirp with 40 ms pulse width and 20 ms slice width for reconstructions. The dashed black lines indicate the regions where the cross-sectional cuts (displayed underneath the whole volume) were made. The reconstructions were generated by linearly rescaling each tomographic slice between (0,1) and stacking the layers. Volumes are displayed using software available online [31].

The eTC-PCT amplitude reconstructions of the lesions are shown in Figure 6. The reconstructions demonstrate the high degree of amplitude channel specificity for the detection of caries, as it is strongly influenced by the change in optical properties causing the increase in thermophotonic heat (thermal infrared photon) generation in the lesions. In all three amplitude reconstructions, the lesions are seen throughout all the slices. This is expected as the amplitude channel is strongly influenced by the radiative thermal component of the thermal transients within the sample. This means that as the thermal centroid moves deeper into the enamel, the re-emission of thermal photons will strongly contribute to the amplitude channel. This helps boost the SNR of defective regions but suppresses the conductive portion of the thermal signals, which is believed to be more revealing of the true morphology of the sample [14]. This is consistent in the TC-PCT amplitude channel as well (not shown in Figure 6), which differs in that it does not contain the quadrature amplitude component (which comes from a later delay time than the in-phase portion as there is no signal truncation), thereby helping improve the localization of the thermal waves [13]. The amplitude tomographic slices (sample results for the buccal surface are provided in the Supplementary Materials: Figure S1) from the two TC-PCT and eTC-PCT algorithms showed minimal difference, producing nearly identical amplitude reconstructions, supporting the findings that the Q component offers little value to the amplitude channel [13].

**Figure 7:**
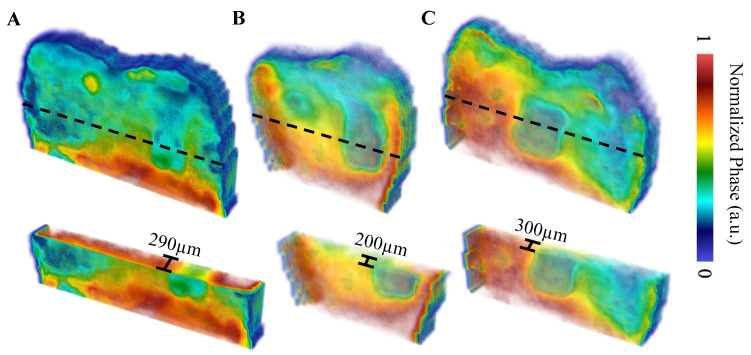
TC-PCT LIOP reconstructions with partial transparency of the (**A**) buccal, (**B**) distal, and (**C**) lingual surfaces using a 0.2–0.6 Hz, 12 s chirp with 40 ms pulse width and 20 ms slice width. All reconstructions were filtered using the histogram equalization filter [13] to improve the caries visibility. The dashed black lines indicate the regions of the cross-sectional cuts (displayed underneath the whole volume). Volumes are displayed using software available online [31].

The TC-PCT LIOP volume reconstructions are shown in Figure 7. The lesions are detected in all three reconstructions, albeit with less visibility than the amplitude channels. This is due to division of the quadrature signal by the in-phase signal, which helps normalize the depth-integrated radiative thermal emission component of the thermal transients. All the TC-PCT LIOP reconstructions were enhanced using a slice-by-slice global histogram equalization algorithm [13]. This drastically improved the visibility of the subsurface defects by uniformly distributing the pixel intensity histograms of the reconstructions, in turn, improving the defect contrast while preserving spatial coherence. The TC-PCT LIOP reconstructions show the lesions within the first slice of the reconstructions with a slow emergence of the apparent end of the lesions in the distal (Figure 7B) and lingual (Figure 7C) reconstructions. This occurs as the thermal transients from the lesions and sound enamel equalize in depth, causing the loss in contrast. This does not occur in the buccal reconstruction (Figure 7A), possibly indicating that at the latest delay time, or final slice, the carious and sound enamel carry thermal transients originating from differing depths. Alternatively, differences in subsurface thermal properties may be responsible for the difference in thermal decay seen in these regions, even when the thermal transients are generated at the same depth. These LIOP reconstructions are consistent with the µ-CT measurements (Figure 3), showing that the lingual and buccal surface lesions have the greatest depth of penetration (300 µm and 290 µm, respectively), followed by the distal surface lesion (200 µm). However, for the chosen chirp duration (12 s), the reconstructions are unable to resolve the end of the caries for the buccal surface, despite doing so for the slightly deeper lingual surface lesion. This highlights the difficulty in producing true quantitative measurements of lesion depth using TC-PCT and its analogues, due to the complex multilayered nature and high variation in optical and thermal properties amongst different teeth and surfaces.

**Figure 8:**
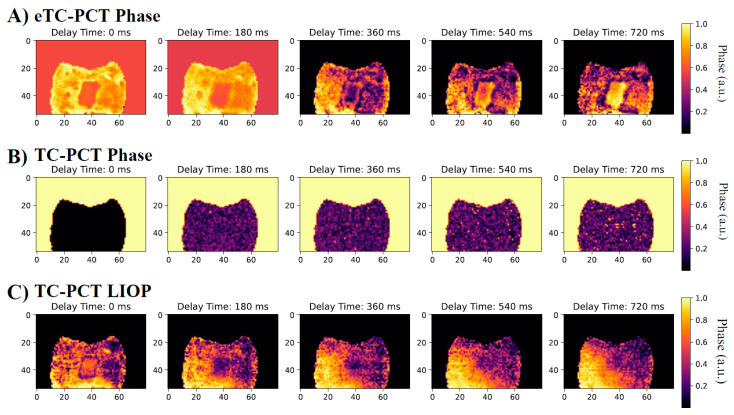
Select tomographic phase slices: (**A**) eTC-PCT phase, (**B**) TC-PCT phase, and (**C**) TC-PCT LIOP of the B8 sample lingual surface after 8 days of bacteria-induced demineralization. Their corresponding phase values were normalized between 1.0 and 0 by subtracting the minimum phase value and then dividing by the maximum phase value within each slice. All slices were calculated using the same data from a 0.2–0.6 Hz, 12 s chirp with 63 W peak laser power. Contrast of the slices has been enhanced using the slice-by-slice global histogram equalization algorithm described in software available online [13].

Select tomographic slices of the lingual surface are shown in Figure 8 for the eTC-PCT phase (Figure 8A), the TC-PCT phase (Figure 8B), and the LIOP (Figure 8C). The eTC-PCT phase exhibits the highest contrast between the lesions and the surrounding healthy enamel, whereas TC-PCT is unable to clearly detect the lesion. This is due to the high noise levels in the TC-PCT phase, which requires high SNR thermal signals for quality reconstructions; this is not feasible when imaging teeth, due to the low NIR optical absorption in healthy enamel and the MPE restrictions limiting the optical pulse power [24]. The TC-PCT LIOP detects the lesion in the initial slices, but the contrast quickly disappears as the LIOP sound and carious transient evolution equalizes, allowing it to produce the volumetric reconstruction with the isolated caries seen in Figure 7C. Additional investigation is needed to improve the quantitative understanding of the thermal transient decay rates to estimate the true depths of the lesions.

### 3.2. Samples M: 2-, 4-, 6-, and 8-Day Bacterial Demineralization Treatments

**Figure 9:**
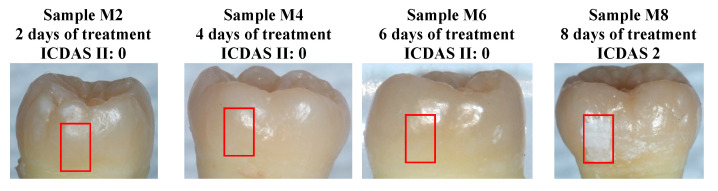
Optical images of samples M2, M4, M6, and M8 after cariogenic biofilm exposure. The red rectangles show the regions of exposure on the teeth. Only sample M8 (8 days of cariogenic biofilm exposure) developed visible caries.

Four separate samples, M2, M4, M6, and M8, were treated for 2, 4, 6, and 8 days each, respectively, using the bacterial exposure protocol outlined in Section 2.1. Optical images of the four treated surfaces are shown in Figure 9, with red boxes indicating the treatment windows used for bacterial exposure. Lesions on samples M2, M4, and M6, ranked as ICDAS 0 at the sites of exposure, meaning that the enamel was scored as “Sound” through visual inspection. In sample M8, the treated region developed an ICDAS 2 lesion at the site of exposure, corresponding to a “distinct visual change in enamel” according to the ICDAS criteria.

**Figure 10:**
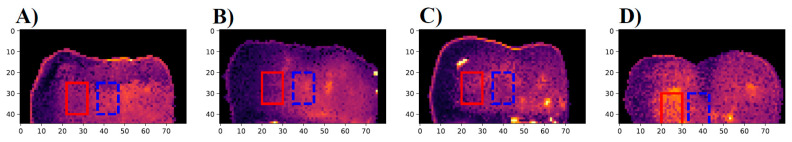
First slice of the eTC-PCT amplitude for samples (**A**) M2, 2 days of treatment, (**B**) M4, 4 days of treatment, (**C**) M6, 6 days of treatment, and (**D**) M8, 8 days of treatment. All images were acquired using a 0.2–0.6 Hz, 12 s chirp with 40 ms pulse widths, 63 W peak laser power and 20 ms slice widths for reconstructions. The red rectangle highlights the treatment region where the samples were exposed to the cariogenic biofilm. The dashed blue rectangle highlights the nearby sound region that was not exposed to the cariogenic biofilm. This region was used to determine a baseline sound enamel signal.

**Figure 11:**
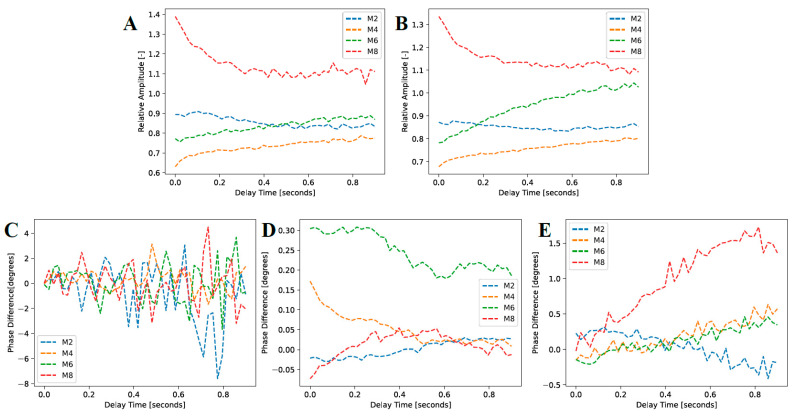
Mean amplitude and phase responses of the caries lesions and sound enamel regions highlighted in Figure 10 for (**A**) TC-PCT amplitude, (**B**) eTC-PCT amplitude, (**C**) TC-PCT phase, (**D**) eTC-PCT phase, and (**E**) TC-PCT LIOP.

The first eTC-PCT amplitude slices for the M2, M4, M6, and M8 samples are shown in Figure 10. Only the lesion on the M8 sample (8 days of cariogenic biofilm exposure) is clearly visible in the slices. To better understand the photothermal dynamics associated with imaging of the treated regions, the relative amplitude and phase differences are plotted in Figure 11, which were calculated using Equations (1) and (2), respectively.

(1) $$Relative\;Amplitude\left(\tau \right)=\frac{Treated\;Amplitude\left(\tau \right)}{Sound\;Amplitude\left(\tau \right)}$$

(2) Phase Difference(τ)=Treated Phase(τ)−Sound Phase(τ)

Here, τ is the delay time, and the “Treated Amplitude” and “Sound Amplitude” are the mean amplitude responses of the treated and sound regions, respectively (locations are outlined in Figure 10). The “Treated Phase” and “Sound Phase” are the mean phase responses from the same regions. This is determined rather than looking at the absolute values of the amplitude and phase channels to help normalize signal variations between samples with differing structures and optical and thermal properties. In addition, the contrast between caries lesions and sound enamel is more critical than their absolute values, as this dictates the empirical visibility of the caries lesions. As seen in Figure 11A,B, the TC-PCT and eTC-PCT amplitude channels, respectively, show strong relative absorption in the M8 sample. Again, this is caused by an increase in optical scattering and absorption coefficients in the caries lesion. Samples M2, M4, and M6 all show approximately constant relative amplitudes, as there is no significant difference in photon absorption between the treatment regions and reference sound enamel, indicating that no detectable lesion was formed. These short periods of treatment time (M2, M4, and M6) even show a relative absorption under 1.0, which is likely due to a combination of natural optical property variation within the enamel and uneven reflectance due to the curved surface of the samples, causing optical intensity inhomogeneity over the sample surfaces.

The TC-PCT phase difference (Figure 11C) is noisy in all four samples, fluctuating about 0°, showing no real discernible trend. Again, this is the result of the low heat generation and concomitant low SNR in the enamel due to low absorption and MPE limitations. For the eTC-PCT phase ( Figure 11D), the M4 and M6 samples show a decrease in the phase difference. This is explainable by increased scattering in the demineralized region, attenuating the emission of near-subsurface thermal photons, causing conductive heat transfer from deeper regions to dominate the thermal response. This results in a net positive phase difference that decreases with elapsed time as deeper subsurface regions in the reference sound enamel become thermally involved. Sample M6 exhibits this conductive-dominant behavior most clearly, with M4 demonstrating it to a lesser extent. However, the M2 sample’s eTC-PCT phase difference is nearly a constant zero, indicating very similar optical and thermal properties between the treated and baseline regions. The M8 sample with the most evident demineralization (ICDAS 2) behaves differently. The eTC-PCT phase is initially negative due to the highly scattering caries lesion causing strong near-surface energy localization and confinement within the thin demineralized layer, and prevention of bulk thermal photons from being clearly emitted. The phase difference then gradually increases to a near-zero positive value as some heat from the confined surface absorption region diffuses inward, and the thermal diffusion centroid converges to that of the sound enamel.

The TC-PCT LIOP, as presented in Figure 11E, also demonstrates trends for in M4, M6, and M8 samples that are consistent with increased scattering in the enamel. All three samples exhibit an increasing LIOP with delay time as the early demineralization in M4, M6, and M8 causes increased photon scattering of the laser excitation source, gradually blocking the bulk generation and emission of thermal photons. The optical-to-thermal conversion of incident photon energy generates heat confined in the bacterial exposure region, conductively traveling out into the bulk at a greater flow rate due to the large surface thermal confinement (lower thermal diffusivity and reduced photon penetration depth), causing the increase in phase lag relative to the sound regions of enamel. In the LIOP modality, the phase lag of the most demineralized sample, M8, diverges away from those of the other less demineralized samples, as expected, due to the largest near-surface thermal confinement, thereby demonstrating the enhanced sensitivity of this modality.

**Figure 12:**
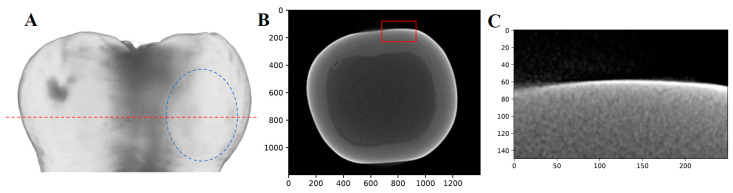
µ-CT results of sample M8 (8 days of demineralization): (**A**) Volume reconstruction with no visible lesion. The dashed blue oval indicates the region where the lesion is visible with the naked eye, the dashed red line indicates the plane where the transverse view B is taken. (**B**) Transverse view of the sample with the red box indicating where the lesion should be visible. (**C**) Enlarged view of the red box seen in B, with no lesion visible.

To further quantify the severity of the lesion on M8, µ-CT was employed; however, the lesion was not detected (the results are shown in Figure 12). This suggests that the lesion, although easily observed with the naked eye, had not progressed significantly into the enamel to be discernible by radiographs. Typically, µ-CT is relatively insensitive to caries lesions that are limited to early caries within the enamel [32], which emphasizes the difficulty in detecting caries early in their formation and the potential sensitivity advantage of TC-PCT thermophotonic imaging.

**Figure 13:**
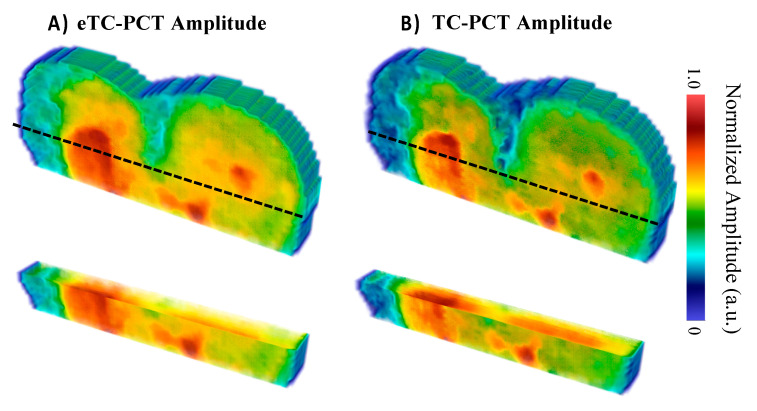
Amplitude reconstructions of the M8 sample (8 days of cariogenic biofilm exposure) using the (**A**) eTC-PCT and (**B**) TC-PCT algorithms with 20 ms slice widths. The amplitudes were normalized between 1.0 and 0 by subtracting the minimum amplitude value and then dividing by the maximum amplitude value within each slice. Volumes are displayed using the method of [31].

The photothermal amplitude reconstructions of sample M8 shown in Figure 13 support the foregoing shallow lesion hypothesis, as the lesion does not extend through all the slices, indicating that there was relatively little heat generated within the caries lesion due to it being very thin (likely under 200 µm, which was detectable on the B8 sample using the same µ-CT imaging setup), allowing the heat to dissipate quickly. In contrast, the amplitude reconstructions for the B8 sample (Figure 6) show the lesions continuously extending throughout all amplitude slices, suggesting greater optical absorption and thermal emission relative to the surrounding sound enamel when compared to the M8 sample. The higher severity of caries in sample B8 is supported by µ-CT, which detected all the lesions, whereas the lesion of M8 was not detected by µ-CT.

The eTC-PCT lesion reconstruction (Figure 13A) presents itself as a shallower and more uniform surface absorption defect in comparison with the TC-PCT reconstruction (Figure 13B). These minor differences are the result of the quadrature contribution to the eTC-PCT amplitude, which is excluded in the TC-PCT amplitude. Due to the rapid decay in the detected infrared signal, the quadrature only contributes strongly to the initial slices, but then is quickly dominated by the in-phase portion of the infrared signal. This results in a more visible surface lesion, suggesting that the inclusion of the Q signal channel may be useful in improving the detection sensitivity of the eTC-PCT and TC-PCT amplitude channels to minor surface caries.

The phase images (TC-PCT, eTC-PCT, and TC-PCT LIOP) were unable to definitively resolve the M8 lesion (images shown in Figure S2 of the Supplementary Materials) as the heat generation was very low. Without the SNR enhancement coming from the radiative component, the phase channels have lower detection sensitivity. This suggests that for very shallow caries, the amplitude channels are preferable.

## 4. Conclusions

In conclusion, dental caries was induced by cariogenic bacteria on extracted human teeth in a controlled manner. Detectable caries lesions, based on thermophotonic imaging, visual assessment, and µ-CT, formed after 8 days of treatment. In addition, thermophotonic imaging of the teeth after 4 and 6 days showed phase and amplitude trends consistent with increased scattering caused by enamel demineralization, but was unable to show definitive volumetric reconstructions of the very early demineralization (ICDAS 0) due to the extreme near-surface localization of the lesion compared to the longer optical penetration depth of the incident laser beam. The TC-PCT, eTC-PCT, and TC-PCT LIOP modalities and algorithms, while operating at clinically safe energy levels, were investigated, and were found to have greater sensitivity to the caries early in their formation than the adopted µ-CT gold standard of in vitro dental imaging. The recently introduced TC-PCT LIOP channel demonstrated excellent three-dimensional capabilities, accurately reconstructing lesions 200–300 µm deep, demonstrating promise as an effective tool for the early detection and quantification of dental caries severity. Additionally, the TC-PCT and eTC-PCT amplitude channels demonstrated greater sensitivity to the caries early in its formation than their phase counterparts, due to the greater SNR of the former channels. Due to the limited number of samples (*n* = 5), statistically significant results could not be produced to support the clinical use of the present non-invasive thermophotonic imaging modality. In the future, additional studies using larger sample sizes of bacteria-induced and natural caries are required to support the clinical adoption of thermophotonic imaging for early-stage dental caries detection.

## Figures and Tables

**Table 1:** ICDAS scoring system used to relate visual appearance of caries to severity [26].

Rank	Title 2
0	Sound
1	First visual change in enamel
2	Distinct visual change in enamel
3	Localized enamel breakdown
4	Underlying dark shadow from dentin
5	Distinct cavity with visible dentin
6	Extensive distinct cavity with visible dentin

## Data Availability

The data presented in this study are available on request from the corresponding author.

## Supplementary Materials

The following supporting information can be downloaded at: https://www.mdpi.com/article/10.3390/bioengineering10010112/s1, Figure S1: Select tomographic amplitude slices: (Top Row) eTC-PCT amplitude and (Bottom Row) TC-PCT amplitude of the B8 sample buccal surface after 8 days of bacterial-induced demineralization. Figure S2: Select tomographic phase slices: (Top Row) eTC-PCT phase and (Middle Row) TC-PCT phase and (Bottom Row) TC-PCT LIOP of the M8 sample surface after 8 days of bacterial-induced demineralization.
