# Supplementary material for: Detection of Bacteria-Induced Early-Stage Dental Caries Using Three-Dimensional Mid-Infrared Thermophotonic Imaging
Source: Bioengineering (Basel). 2023 Jan 12;10(1):112. doi: 10.3390/bioengineering10010112 (PMC9854787; doi:10.3390/bioengineering10010112)
Supplement: Supplementary file 1 [file bioengineering-10-00112-s001.zip › bioengineering-2106999-supplementary.pdf]

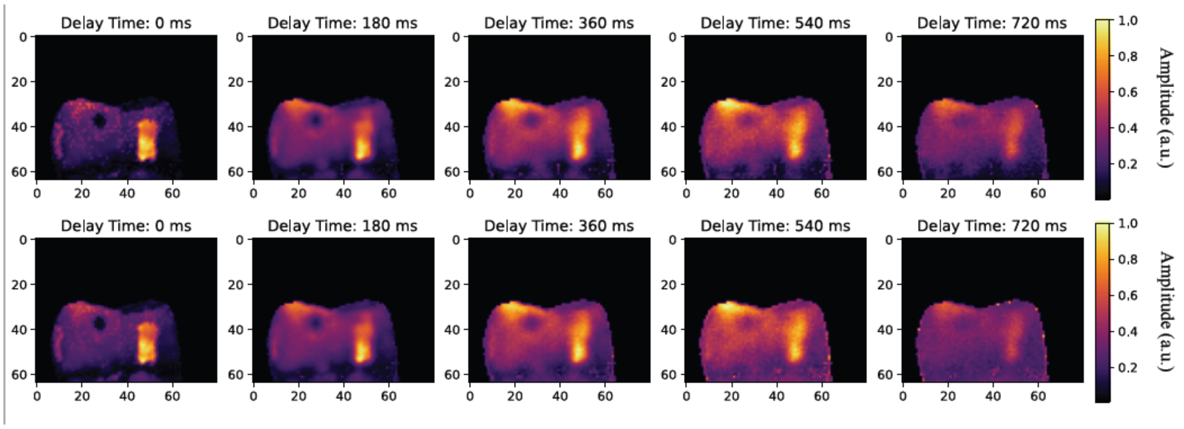

Figure S1. Select tomographic amplitude slices: **(Top Row)** eTC-PCT amplitude and **(Bottom Row)** TC-PCT amplitude of the B8 sample buccal surface after 8 days of bacterial-induced demineralization. Their corresponding amplitude values were normalized between 1.0 and 0 by subtracting the minimum amplitude value and then dividing by the maximum amplitude value within each slice. All slices were calculated using the same data from a 0.2-0.6 – Hz, 12-s chirp with 63-W peak laser power. Contrast of the slices has been enhanced using the slice-by-slice global histogram equalization algorithm described in software available online [13].

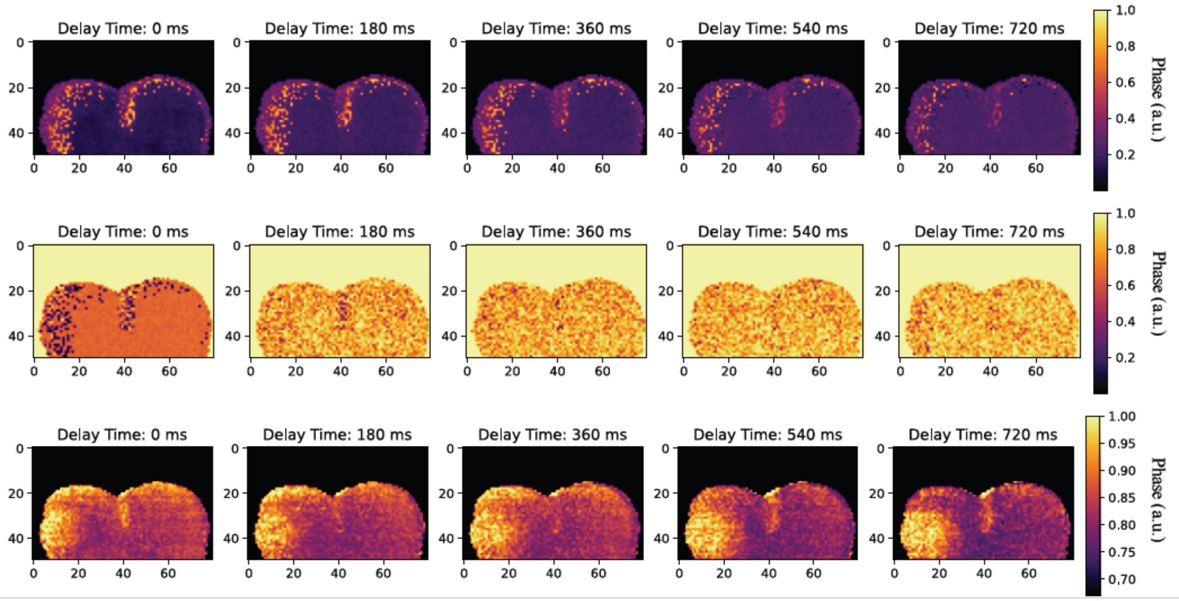

Figure S2. Select tomographic phase slices: **(Top Row)** eTC-PCT phase and **(Middle Row)** TC-PCT phase and **(Bottom Row)** TC-PCT LIOP of the M8 sample surface after 8 days of bacterial-induced demineralization. The caries lesion is not visible in any of the phase images. Their corresponding phase values were normalized between 1.0 and 0 by subtracting the minimum phase value and then dividing by the maximum phase value within each slice. All slices were calculated using the same data from a 0.2-0.6 – Hz, 12-s chirp with 63-W peak laser power. Contrast of the slices has been enhanced using the slice-by-slice global histogram equalization algorithm described in software available online [13]
